# Governing Generative AI in Healthcare: A Normative Conceptual Framework for Epistemic Authority, Trust, and the Architecture of Responsibility

**DOI:** 10.3390/healthcare14081098

**Published:** 2026-04-20

**Authors:** Fatma Eren Akgün, Metin Akgün

**Affiliations:** 1 Elderly Care Programme, Department of Health Care Services, Vocational School of Health Services, Ağrı İbrahim Çeçen University, 04100 Ağrı, Türkiye; f.eren1789@hotmail.com; 2 Department of Pulmonary Diseases, School of Medicine, Ağrı İbrahim Çeçen University, 04100 Ağrı, Türkiye; 3 Department of Systematic Philosophy and Logic, Faculty of Letters, Atatürk University, 25240 Erzurum, Türkiye

**Keywords:** generative artificial intelligence, healthcare, large language model, epistemic authority, warranted trust, responsibility gap, automation bias, patient safety, AI governance, human oversight

## Abstract

**Highlights:**

**What are the main findings?**

- Healthcare AI governance requires classifying LLM outputs into distinct tiers—from administrative drafts to clinical evidence claims—each with appropriate verification.
- Generic ‘human oversight’ declarations can function as ‘epistemic placebos’ that create the appearance of safety without genuine safeguards.

**What are the implications of the main findings?**

- Healthcare institutions need explicit governance frameworks that match oversight intensity to the clinical risk level of each AI deployment.
- The 2025–2027 regulatory transition period is the critical window for establishing these norms before institutional defaults are set by vendor design.

**Abstract:**

Background/Objectives: Large language models (LLMs) such as ChatGPT are rapidly being integrated into healthcare for tasks ranging from clinical documentation to diagnostic support. Current ethical discussions focus predominantly on bias, privacy, and accuracy, leaving three critical governance questions unresolved: What kind of knowledge does an LLM output represent in clinical reasoning? When is a clinician’s or patient’s trust in that output justified? Who bears responsibility when an AI-informed decision leads to patient harm? This study proposes the Epistemic Authority–Trust–Responsibility (ETR) Architecture, a normative conceptual framework that addresses these three questions as an integrated governance challenge. Methods: The framework was developed through normative conceptual analysis—a method that constructs governance proposals by synthesising philosophical principles, ethical theories, and empirical evidence. The literature was identified through structured searches of PubMed, PhilPapers, and EUR-Lex (January 2020–March 2026), drawing on the philosophy of medical knowledge, the ethics of trust and testimony, and the moral philosophy of responsibility. Results: The ETR Architecture produces four outputs: (i) a four-tier classification system that distinguishes LLM outputs—from administrative drafts to clinical evidence claims—and matches each tier to appropriate verification requirements; (ii) the concept of the ‘epistemic placebo’, formally defined as a governance measure that creates a documented appearance of compliance while lacking at least one operative element of genuine oversight; (iii) a model specifying four conditions under which trust in healthcare AI is justified; (iv) four testable hypotheses with associated research designs connecting governance design to trust calibration and patient safety. Conclusions: The 2025–2027 regulatory transition period offers a critical window for shaping how healthcare institutions govern AI. We argue that deploying LLMs without explicitly classifying their outputs and building appropriate oversight risks allows governance norms to be set by technology vendors rather than by evidence-informed, patient-centred policy.

## 1. Introduction

When a large language model (LLM) such as ChatGPT drafts a discharge summary, suggests possible diagnoses, or replies to a patient’s message on behalf of a clinician, it is doing more than saving time. It is producing content that looks and reads like medical knowledge, and patients and clinicians may treat it as such [1]. This raises questions that healthcare institutions have not yet answered: Is an LLM’s output a piece of evidence, a suggestion, or simply a rough draft? When should a clinician trust it? And if something goes wrong, who is responsible—the doctor, the hospital, or the company that built the model? These are not abstract concerns. A systematic review of 4609 peer-reviewed studies evaluating LLMs in clinical medicine (2022–2025) found that governance-related outcomes—including epistemic classification, trust calibration, and responsibility attribution—were addressed in fewer than 3% of included studies [2]. International bodies have recognised this gap: the WHO has issued specific governance guidance for large AI models [3], and analyses have warned of automation bias risks in AI-driven clinical decision support [4].

The ethical discussions that do exist have focused mainly on bias, privacy, and accuracy [5,6]. These are important issues, but they miss a more fundamental problem: they assume that the LLM is only a tool, when in practice it is increasingly being treated as a source of clinical knowledge. Consider the available evidence: A large benchmarking study tested 20 LLMs with prompts containing false medical information and found that 31.7% of the time, the models accepted the misinformation as true. When the same false claims were written in the formal language of hospital discharge notes, the acceptance rate rose to 46.1% [7]. These findings suggest that the more an LLM output resembles authoritative medical text, the more difficult it becomes—even for the model itself—to identify potential errors. Meanwhile, a randomised trial showed that giving doctors access to an LLM did not actually improve their diagnostic accuracy [8]. If LLMs can be susceptible to clinical-sounding misinformation and do not demonstrably improve diagnostic accuracy, then the way by which we govern their use in healthcare needs rethinking.

This essay proposes a normative governance framework—the Epistemic Authority–Trust–Responsibility (ETR) Architecture—designed to help healthcare institutions answer three questions before deploying an LLM: (1) What type of output is this system producing, and what level of verification does it require? (2) Under what conditions should clinicians and patients trust its outputs? (3) Who is accountable if the output contributes to patient harm? The framework is particularly timely because the EU Artificial Intelligence Act, which classifies certain medical AI systems as ‘high risk’ under Annex III, will become fully applicable between 2026 and 2027 [9,10], and the WHO has issued specific governance guidance for large AI models in healthcare [11,12]. Other jurisdictions—including the United States, China, and Brazil—are developing their own approaches. The governance norms that healthcare institutions establish now during this transitional period will shape clinical AI practice for years to come.

### 1.1. Contributions and Scope

The existing literature addresses the epistemic status of AI in clinical settings [13], trust calibration in healthcare AI [14,15,16], and responsibility distribution [17,18,19,20,21] as separate concerns. The ETR framework’s distinctive contribution is to integrate these three dimensions into a single governance architecture with operational specificity—providing not only conceptual analysis but also decision criteria, responsible actors, and documentation requirements for each governance function. The framework operates at the institutional governance layer, between study-level evaluation standards (DECIDE-AI [22] and TRIPOD+AI [23]) and high-level regulatory requirements (EU AI Act [9,10] and WHO guidance [11,12]), filling a gap that neither principled ethics frameworks nor technical standards currently address.

This essay provides five contributions: (1) it proposes a four-tier classification system for LLM outputs in healthcare—from simple drafts to clinical evidence claims—each matched to the level of verification it requires, with operational decision criteria and responsible actors; (2) it introduces the concept of the ‘epistemic placebo’ to describe governance measures that appear reassuring but do not actually protect patients, with a formal definition and empirically identifiable markers; (3) it identifies four conditions that must be met before trust in a healthcare LLM is justified; (4) it proposes a structured model for distributing responsibility across developers, hospitals, clinical teams, and auditors (RACI model); (5) it generates four testable hypotheses with associated research designs that future studies can use to validate or refute the framework.

### 1.2. Methodological Approach

This framework was developed through normative conceptual analysis—a method that constructs governance proposals by synthesising philosophical principles, ethical theories, and empirical evidence rather than by inductively deriving recommendations from a single data source [24]. The methodological approach comprised three phases.

Phase 1—Literature identification: Sources were identified through structured searches of PubMed (empirical and clinical literature), PhilPapers (philosophical works), and EUR-Lex and WHO IRIS (regulatory and policy documents), covering January 2020–March 2026 and supplemented by backward citation tracking and targeted expert recommendation. Sources were included if they addressed the following: (i) the epistemic status of AI outputs in clinical contexts, (ii) trust dynamics in human–AI clinical interaction, (iii) responsibility attribution in AI-mediated healthcare decisions, or (iv) regulatory frameworks governing healthcare AI. Sources addressing AI in non-healthcare domains were excluded unless they offered transferable governance insights.

Phase 2—Convergent conceptual analysis: Three bodies of scholarship were analysed for governance-relevant principles. From the philosophy of medical knowledge [13], we derived the epistemic classification of outputs (the four-tier system). From the ethics of trust and testimony [14,15,16], we derived the conditions for warranted trust. From the moral philosophy of responsibility [17,18,19,20,21], we derived the prospective responsibility allocation model. The ETR framework’s components were constructed at the intersection of these three domains.

Phase 3—Integration with empirical evidence and regulatory context: The conceptual components were contextualised against recent empirical findings (e.g., LLM susceptibility to misinformation [7], diagnostic accuracy trials [8]) and current regulatory developments (EU AI Act [9,10], WHO guidance [11,12], and NIST AI RMF Generative AI Profile [25]). Empirical evidence is used illustratively to motivate and contextualise the normative framework rather than to inductively derive it.

## 2. The ETR Framework

**Figure 1:**
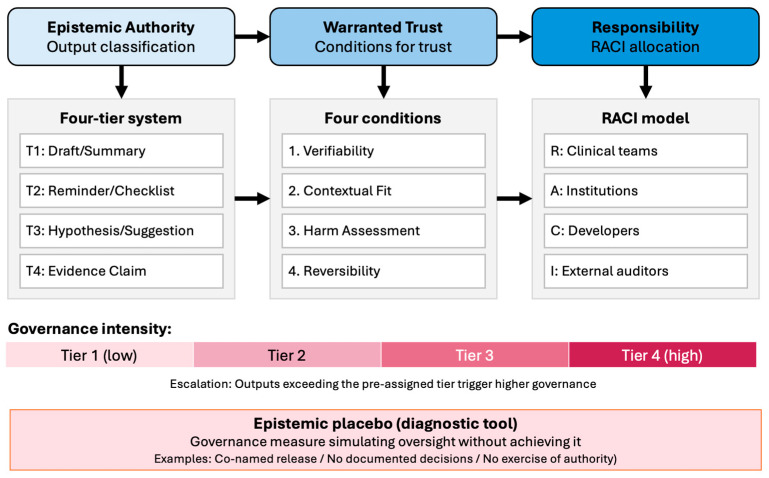
The Epistemic Authority–Trust–Responsibility (ETR) Architecture. The framework integrates three governance dimensions: epistemic classification (four-tier system, left), warranted trust (four conditions, centre), and responsibility allocation (RACI model, right). The epistemic placebo concept (bottom) identifies governance measures that simulate oversight without achieving it. Governance intensity increases from Tier 1 (administrative drafts) to Tier 4 (evidence claims). Arrows indicate the flow from output classification through trust assessment to responsibility assignment.

Figure 1 provides a synthetic overview of the ETR Architecture, illustrating how its three core dimensions—epistemic authority (output classification), warranted trust (conditions for justified reliance), and responsibility (RACI allocation)—interact within a unified governance structure. The figure also shows the role of the epistemic placebo concept as a diagnostic tool for identifying governance failures, and it indicates how governance intensity scales with the tier level of LLM outputs.

### 2.1. Epistemic Authority and the Status of LLM Outputs: A Medical–Philosophical Analysis

In medicine, not all information carries the same weight. A randomised controlled trial is treated differently from a case report; a specialist’s opinion is treated differently from an automated alert. This is not arbitrary—it reflects how each type of information is produced, how rigorously it has been tested, and how much confidence clinicians can reasonably place in it [13]. LLMs produce text by predicting the most likely next word based on patterns in their training data. This process is fundamentally different from the cause-and-effect reasoning that underpins clinical medicine. An LLM can generate a sentence that reads like a guideline recommendation without any of the evidence review, peer scrutiny, or clinical validation that would normally stand behind such a statement.

Recent philosophical work has argued that medical AI should be understood not as a rival to the doctor’s expertise but as a tool that changes how knowledge is produced and used in clinical settings [13]. We agree but argue that this insight alone is not enough for practical governance. The real question facing hospitals and clinicians is this: What kind of output is this LLM actually producing, and what verification does it need? To answer this, the ETR framework proposes a four-tier classification. At Tier 1, the LLM produces a draft or summary—for example, a referral letter assembled from the patient’s record—which needs clinician review before being sent. At Tier 2, it generates a reminder or checklist item—such as flagging that a patient on long-term steroids has not had a bone density scan—which needs a traceable source and a log of whether the clinician acted on it. At Tier 3, it offers a hypothesis or suggestion—such as a list of possible diagnoses for a patient with unexplained lung disease—which requires independent clinical validation and a documented reason for accepting or rejecting it. At Tier 4, it makes an evidence claim—such as stating that two drugs are contraindicated based on a specific interaction—which demands a traceable citation to primary evidence and a formal institutional audit. The critical distinction is that a hospital treating all LLM outputs identically—labelling everything ‘clinical decision support’ without distinguishing a draft from an evidence claim—is relying on unstructured clinical judgement without institutional governance support.

Tier assignment is an institutional pre-deployment decision. The healthcare institution’s AI governance committee assigns a tier based on the system’s intended use and maximum output risk level. When a system’s output shifts to a higher tier during use (e.g., a Tier 1 drafting tool begins generating diagnostic suggestions), the governance requirements of the highest tier reached apply to the entire interaction. Institutions must define escalation triggers in their deployment protocols.

Specific escalation triggers should include the following: (a) output content triggers—the system generates content characteristic of a higher tier (e.g., a Tier 1 drafting tool produces a statement beginning ‘Based on clinical evidence…’ or ‘This drug is contraindicated with…’); (b) user interaction triggers—a clinician poses a query that elicits a higher-tier response (e.g., asking a summarisation tool ‘What diagnosis fits these symptoms?’); and (c) system behaviour triggers—the model’s response pattern shifts from its validated use case (e.g., generating unsolicited differential diagnoses within a documentation workflow). When any trigger is detected, the governance requirements of the higher tier apply immediately, the interaction is flagged in the audit trail, and the clinical team is alerted to apply the verification protocol corresponding to the escalated tier.

### 2.2. Warranted Trust: From the Epistemology of Testimony to Healthcare AI Ethics

Trust in healthcare AI is not only a matter of whether people feel comfortable using it. The more important question is whether that trust is justified—whether the conditions that would make reliance reasonable are actually in place [14]. A systematic review of healthcare workers’ attitudes found that trust in AI decision support depends heavily on the institutional and technical context in which it is used, not on general assurances of safety [15]. Experimental research confirms that what drives trust is not labels or certifications but whether users perceive the system as genuinely competent in their clinical domain [16]. In short, telling clinicians ‘this system has been approved’ is far less effective than showing them how and why it works in their specific setting.

Based on this evidence, the ETR framework identifies four conditions that must be considered before trust in a healthcare LLM is warranted. The first is verifiability: Can the output be checked against a known source? If an LLM states that a drug is contraindicated, it should be possible to trace that claim to a specific guideline or study. The second is contextual fit: Does the level of oversight match the level of clinical risk? A Tier 1 draft needs a quick review; a Tier 4 evidence claim needs a formal audit. The third is harm assessment: Has someone explicitly evaluated what could go wrong if this particular output is incorrect [4]? Finally, the fourth is reversibility: If the AI-informed decision turns out to be wrong, can it be undone or corrected? A misdirected referral letter is easy to fix; an inappropriate treatment recommendation may not be. Not all four conditions carry equal weight in every situation—for a simple draft, contextual fit matters most; for an evidence claim, verifiability is paramount.

These conditions expose a problem with how ‘human oversight’ is currently understood in healthcare AI governance. Many institutions and regulations require human oversight without specifying what that means in practice: Who reviews the output, at what point, and with what authority to override? When ‘human oversight’ exists on paper but lacks a defined reviewer, a clear process, and real consequences for non-compliance, it becomes what we call an epistemic placebo. We define this concept formally:

Definition: An epistemic placebo is a governance measure that (i) is presented as providing oversight over AI-generated outputs and (ii) creates a documented appearance of compliance, but (iii) lacks at least one of the following operative elements: (a) a designated reviewer with domain-relevant competence, (b) a specified review process with defined decision points, (c) enforceable consequences for non-compliance, or (d) an auditable record of review actions and outcomes.

Boundary condition: Partial oversight—where some but not all operative elements are present, and the gaps are documented and scheduled for remediation—does not constitute an epistemic placebo, provided that the institution has an explicit, time-bound plan for achieving full compliance. The epistemic placebo is distinguished from ‘ethics washing’ (public-facing ethical claims without substantive commitment) and from ‘security theatre’ (measures designed for appearance rather than effectiveness) by its specific focus on oversight mechanisms that simulate epistemic verification without achieving it. In practice, auditors can detect an epistemic placebo by checking for three observable indicators: absence of a named, qualified reviewer; absence of documented review decisions (accept/reject/modify); and absence of enforceable override authority.

The epistemic placebo is particularly dangerous for three reasons: It mimics real governance, so that institutions believe they are protected. It can exploit regulatory language (‘AI Act compliant’) to create a false sense of security [9]. It is self-reinforcing—once an institution believes that it has ‘ticked the oversight box’, it has less incentive to invest in genuine safeguards.

### 2.3. The Responsibility Gap: From Moral Philosophy to Healthcare Governance Design

When an AI-informed clinical decision results in patient harm, a straightforward question arises: Who is responsible? In traditional medicine, the answer is relatively clear—the treating clinician, the institution, or both. However, when an LLM generates a recommendation that a clinician follows, the picture becomes more complicated. The developer built the model but did not see the patient. The clinician saw the patient but may not understand how the model generated its output. The hospital purchased the system but may not have the expertise to evaluate it. This is what the literature calls the ‘responsibility gap’ [17,18]. Two solutions have been proposed: distributing responsibility prospectively between all stakeholders [19] or fundamentally rethinking what it means to be a responsible clinician when part of the clinical reasoning is carried out by a machine [20,21].

The ETR framework combines elements of both approaches. It treats responsibility not as a philosophical puzzle to be debated after harm occurs, but as a design problem to be solved before deployment. Six governance functions must each have a clearly assigned owner: model validation (is the model safe for this use?), output classification (what tier is this output?), output verification (has a qualified person checked it?), harm detection (how will errors be caught?), audit trail maintenance (is there a record of who did what?), and model lifecycle management (what happens when the model is updated?). These functions are distributed across four parties—developers, healthcare institutions, clinical teams, and external auditors—using an RACI (Responsible–Accountable–Consulted–Informed) model. Under this model, developers are responsible and accountable for model validation and lifecycle management; healthcare institutions are accountable for output classification, output verification, harm detection, and audit trail maintenance; clinical teams are responsible for output verification and harm detection in daily practice; external auditors provide independent assurance for audit trail maintenance and harm detection. The level of governance is proportionate to the output tier. The key principle is that no function may be left unassigned. If no one is specifically responsible for verifying a Tier 4 evidence claim, this is not a gap to be tolerated; it is a design failure. Current AI ethics auditing practices often miss this point, focusing narrowly on technical metrics such as bias while ignoring whether outputs are appropriately classified and whether trust in them is justified [26].

This model assumes cooperative stakeholder relationships, but real-world deployment involves structural tensions. Developers’ commercial incentives may conflict with institutions’ patient safety obligations; vendors’ superior technical knowledge creates information asymmetry that can constrain institutional governance authority; clinicians’ liability concerns may conflict with institutional efficiency goals. Resource-limited settings—rural clinics and low-income health systems—may lack the personnel for full RACI implementation. We recommend contractual transparency requirements and independent third-party auditing as countermeasures, and we suggest that resource-constrained settings limit LLM deployments to Tiers 1–2 unless full governance infrastructure can be established.

For resource-limited settings, we propose a minimal implementation pathway with three components: (1) Tier restriction—deployments are limited to Tiers 1–2 unless external governance support (e.g., from a referral centre or regional health authority) is available for higher-tier oversight; (2) simplified RACI—in settings without dedicated AI governance officers, the functions of output classification and verification are consolidated under the senior clinician on duty, with institutional accountability retained by the facility administration; and (3) essential documentation—a minimum audit trail is carried out, consisting of a binary log (reviewed/not reviewed) for each LLM-generated output rather than the full verification documentation required in well-resourced settings. This minimal pathway prioritises feasibility over comprehensiveness, but it preserves the framework’s core principle that no LLM output should enter clinical workflows without at least one designated verification step.

### 2.4. Practical Implications for Patient Safety and Healthcare Delivery

What does this mean in practice? Consider a university hospital that deploys the same LLM system in two departments. In one, the LLM drafts after-visit summaries for patients (Tier 1) and generates medication reconciliation checklists (Tier 2). The governance requirement is modest: A clinician reviews each summary before it is released, and an edit trail is kept. In the other department, the LLM suggests possible diagnoses for complex interstitial lung disease cases (Tier 3). Here, the requirement is significantly higher: each suggestion must be independently validated by the treating clinician, with documented reasoning for why it was accepted or rejected.

Suppose that the Tier 3 system generates a drug interaction alert (a Tier 4 evidence claim embedded within a Tier 3 workflow) that a junior clinician accepts without independent verification, leading to an inappropriate medication change. Under the ETR framework, the audit trail would reveal the following: (a) the specific output constituted a Tier 4 evidence claim; (b) the escalation protocol required formal verification, which did not occur; (c) responsibility for output verification rests with the clinical team (responsible) and the institution (accountable) under the RACI model; and (d) the absence of documented verification constitutes a governance failure, not merely an individual clinical error. This example illustrates how the framework shifts adverse event analysis from individual blame to systemic governance evaluation. In the ideal scenario, the drug interaction alert would have triggered an output content escalation (Tier 4 content embedded in a Tier 3 workflow), activating an automated flag requiring senior clinician review and formal verification before any medication change could be implemented.

Matching governance intensity to clinical risk is standard practice in patient safety (e.g., the WHO Surgical Safety Checklist), but this has not yet been systematically applied to AI-mediated clinical workflows. At the institutional level, the framework recommends that hospitals designate specific roles—such as an AI governance officer and a clinical AI oversight committee—with defined accountability for each of the six governance functions. This aligns with the EU AI Act’s requirements for organisations that deploy high-risk AI and with the WHO’s call for independent third-party auditing [9,11,12], but it provides the operational specificity that regulatory language typically lacks.

## 3. Regulatory–Governance Context

The EU AI Act does not classify all healthcare AI as high-risk. Under Annex III, high-risk designation applies to AI systems intended as safety components of, or constituting, medical devices subject to third-party conformity assessment [9,10]. An LLM used solely for administrative drafting (Tier 1) may fall outside this classification, whereas one generating diagnostic suggestions (Tier 3) or evidence claims (Tier 4) is far more likely to meet the threshold. Healthcare institutions must evaluate their specific LLM deployments against the Act’s intended-use criteria rather than assuming blanket high-risk classification [27].

The WHO’s governance guidance has become increasingly specific, identifying risks such as misinformation, automation bias, and cybersecurity threats and calling for independent auditing of healthcare AI systems [11,12]. In the United States, the NIST AI Risk Management Framework and its Generative AI Profile (AI 600-1) provide a complementary, lifecycle-based approach emphasising governance, risk mapping, and measurement [25]. A comprehensive review of AI ethics in healthcare confirms that while there is broad agreement on principles—transparency, accountability, fairness, do no harm—the gap between these principles and their implementation in real clinical settings remains wide [1,28].

The period between 2025 and 2027 is therefore not a pause before regulation takes effect; it is the period during which hospitals, clinics, and health systems are establishing the norms that will govern how they use AI. Institutions that adopt LLMs during this window without a clear governance framework risk having their practices shaped by vendor defaults and clinician improvisation rather than by deliberate, patient-centred design.

### Positioning Within the Healthcare AI Evaluation Ecosystem

The ETR framework is designed to complement, not replace, existing healthcare AI evaluation and reporting standards. DECIDE-AI [22] provides guidance for early-stage clinical evaluation of AI decision support; TRIPOD+AI [23] addresses prediction model reporting; STARD-AI [29] covers diagnostic accuracy reporting. These standards operate at the study level, governing how individual AI systems are evaluated and reported. The ETR framework operates at the institutional governance level, addressing how healthcare organisations classify, verify, and oversee AI outputs in routine clinical practice.

Specific interface points connect the two levels: Tier 3 and Tier 4 outputs should be evaluated using DECIDE-AI criteria before institutional deployment; ongoing performance monitoring should follow TRIPOD+AI reporting standards; diagnostic applications should meet STARD-AI requirements as part of model validation. The ETR framework thus provides the institutional governance layer within which study-level evaluation results are interpreted, acted upon, and audited. Table 1 provides a structured comparison of the ETR framework with these existing standards, identifying the specific dimensions that the ETR framework addresses beyond study-level evaluation.

## 4. Testable Propositions and Research Directions

Four hypotheses emerge directly from the ETR framework—each falsifiable and each connected to a specific research design.

**H1.**  *(Oversight Specificity): Generic ‘human oversight is in place’ statements do not restore epistemic trust in healthcare AI; trust varies significantly with the type of oversight and its fit to the clinical scenario. If falsified—if generic declarations prove as effective as tier-specific, scenario-matched oversight—then the framework’s insistence on contextual fit is unjustified. Research Design: A factorial randomised experiment should be carried out, comparing generic vs. tier-specific oversight instructions on trust calibration and error detection rates among clinicians using simulated LLM outputs.*

**H2.**  *(compliance-induced over-reliance): Regulatory compliance language (‘EU AI Act compliant’ and ‘high-risk certified’) increases trust perception but simultaneously heightens over-reliance risk when misinformation is present. If falsified—if compliance language does not increase over-reliance—then the epistemic placebo concept is empirically empty. Research design: A between-subjects experiment should be carried out, measuring diagnostic accuracy and confidence when clinicians receive LLM outputs labelled as ‘certified’ vs. ‘uncertified’, with embedded misinformation.*

**H3.**  *(epistemic authority as mediator): Perceived epistemic authority mediates the relationship between trust in AI and clinical usage intention [16]. If falsified—if epistemic authority plays no mediating role—then the framework’s central premise that epistemic status drives trust is wrong. Research design: A cross-sectional survey using structural equation modelling should be carried out to test the mediating role of perceived epistemic authority on the trust–usage intention relationship across clinical specialties and institutional contexts.*

**H4.**  *(institutional safeguards): Institutional epistemic safeguards (source requirements, verification layers, and audit trails) attenuate the susceptibility of LLMs to misinformation embedded in clinical authority language [7]. If falsified—if safeguards do not reduce susceptibility—then the framework’s practical promise that governance design can reduce patient harm is unfounded. Research design: A pre–post quasi-experimental study should be carried out in hospitals implementing tiered governance, measuring LLM output accuracy and misinformation susceptibility before and after safeguard deployment.*

A scoping review of the 2021–2026 literature, conducted in accordance with PRISMA-ScR [30] and JBI guidelines [31], would provide the empirical foundation for these studies by mapping how epistemic status and oversight models are currently handled in published studies. Reporting transparency for LLM studies—including model version, date, and prompt design—warrants particular attention given evidence that such reporting is frequently inadequate [32].

## 5. Discussion

The central argument of this essay is that governing AI in healthcare requires answering three questions together, not separately: What kind of output is the system producing? Is trust in that output justified? And who is accountable if it is wrong? The available literature predominantly addresses these questions in isolation [5,6,13,14,15,16,17,18,19,20,21]; the ETR framework proposes that addressing any one without the others produces governance that is incomplete. A system classified as low-risk (Tier 1) with appropriate trust conditions but no assigned responsibility is still ungoverned. A system with clear responsibility allocation but no output classification gives the responsible party no basis for knowing what to check. The three questions form a single governance architecture.

Empirical validation of the ETR framework would require evidence along three measurable dimensions. The first is clinical error reduction: whether tier-matched verification protocols decrease the rate of adverse events attributable to unverified LLM outputs compared with unstructured oversight. The second is trust calibration accuracy: whether clinicians exposed to the four warranted trust conditions demonstrate more appropriate reliance—neither over-trust nor under-trust—as measured by concordance between confidence ratings and actual output accuracy. The third is governance compliance: whether institutional implementation of RACI-assigned responsibilities produces higher rates of documented review actions and traceable audit trails. These indicators map directly onto the four hypotheses proposed in Section 4: H1 and H2 predict improvements in trust calibration, H3 identifies the mechanism (epistemic authority as mediator), and H4 predicts the overall patient safety outcome. Appropriate study designs include factorial randomised experiments (H1 and H2), cross-sectional surveys with structural equation modelling (H3), and pre–post quasi-experimental designs in implementing hospitals (H4).

### 5.1. Counter-Arguments and Risk Trade-Offs

Two objections deserve direct response. First, requiring explicit output classification for every LLM deployment may slow innovation and delay patient access to beneficial AI tools. This trade-off is real, but the alternative—deploying without classification—does not eliminate governance burden; it simply pushes it onto frontline clinicians, who must then improvise safeguards without institutional support. Structured governance distributes the burden more fairly and makes it visible for audit. Second, LLM outputs may shift between tiers within a single interaction—a system might begin by drafting a summary (Tier 1) but slide into making a diagnostic suggestion (Tier 3). The ETR framework addresses this by assigning the tier to the deployment context and not to individual outputs: the institution decides in advance at which tier the system operates and builds its verification processes accordingly. When outputs exceed the pre-assigned tier during use, the escalation pathways apply, and the governance requirements of the highest tier reached govern the entire interaction.

### 5.2. Limitations

This is a normative conceptual framework; its components are proposals based on philosophical analysis and illustrative empirical evidence, not empirically validated recommendations. The four-tier classification, trust conditions, and RACI assignments are structured proposals requiring empirical testing before adoption.

The regulatory analysis focuses on the EU AI Act and WHO guidance. Jurisdictions with fundamentally different regulatory philosophies—such as the market-driven US approach, China’s state-directed model, or Brazil’s rights-based framework—may require substantial adaptation of the governance architecture. The framework treats LLMs as a single category but does not address differences between text-only models and emerging multimodal systems that process images, audio, or video alongside text. The emergence of autonomous AI agents and real-time clinical integration may require a fundamental revision of the tier structure and trust conditions proposed here. The feasibility of implementing the full RACI model will vary substantially between a well-resourced university hospital and a resource-limited primary care setting; we have recommended that such settings limit deployments to Tiers 1–2 unless full governance infrastructure can be established. Finally, LLM technology is evolving so rapidly that any analysis based on current models may need updating as new capabilities and risks emerge [32].

## 6. Conclusions

The regulatory window of 2025–2027 offers healthcare institutions a choice: they can establish explicit governance norms for how LLM outputs are classified, verified, and overseen, or they can allow those norms to be set by default—shaped by vendor design choices and individual clinician improvisation. The ETR Architecture is offered as a normative proposal—a structured starting point for institutional deliberation rather than a validated governance solution, with patient safety at its centre. Future empirical research—particularly testing whether output classification and tier-matched oversight actually improve trust calibration and reduce harm—will be essential for validating, refining, or refuting the framework’s components. The hypotheses and research designs proposed in Section 4 provide a concrete agenda for this empirical programme.

## Figures and Tables

**Table 1:** Structured comparison of the ETR framework with existing healthcare AI evaluation standards.

Dimension	DECIDE-AI [22]	TRIPOD+AI [23]	STARD-AI [29]	ETR Framework
Primary scope	Early-stage clinical evaluation	Prediction model reporting	Diagnostic accuracy reporting	Institutional governance of AI outputs
Level of operation	Study level	Study level	Study level	Institutional level
Output classification	Not addressed	Not addressed	Not addressed	Four-tier system (Tiers 1–4)
Trust conditions	Not addressed	Not addressed	Not addressed	Four conditions for warranted trust
Responsibility allocation	Implicit (investigators)	Implicit (investigators)	Implicit (investigators)	Explicit RACI model across four parties
Audit requirements	Study reporting checklist	Model reporting checklist	Diagnostic reporting checklist	Institutional audit trail for each output
Regulatory alignment	Regulatory-agnostic	Regulatory-agnostic	Regulatory-agnostic	EU AI Act, WHO guidance, NIST AI RMF
Interface with ETR	Tier 3–4 pre-deployment evaluation	Ongoing performance monitoring	Diagnostic application validation	Governance layer interpreting study-level results

Abbreviations: ETR, Epistemic Authority–Trust–Responsibility; DECIDE-AI, early-stage AI decision support evaluation guideline; TRIPOD+AI, prediction model reporting guideline for AI; STARD-AI, diagnostic accuracy reporting guideline for AI; RACI, Responsible–Accountable–Consulted–Informed; EU AI Act, Regulation (EU) 2024/1689; WHO, World Health Organization; NIST AI RMF, NIST AI Risk Management Framework; Tiers 1–4, ETR four-tier LLM output classification system.

## Data Availability

No new data were created or analysed in this study.

## Abbreviations

The following abbreviations are used in this manuscript: LLM: large language model; ETR: Epistemic Authority–Trust–Responsibility; AI: artificial intelligence; EU: European Union; WHO: World Health Organization; RACI: Responsible–Accountable–Consulted–Informed; NIST: National Institute of Standards and Technology.
